# Callose Deposition in Plasmodesmata and Viroid Invasion of the Shoot Apical Meristem

**DOI:** 10.3389/fmicb.2016.00052

**Published:** 2016-02-02

**Authors:** Ricardo Flores

**Affiliations:** Molecular and Evolutionary Plant Virology, Instituto de Biología Molecular y Celular de Plantas, Universidad Politécnica de Valencia-Consejo Superior de Investigaciones CientíficasValencia, Spain

**Keywords:** *Argyranthemum*, callose, chrysanthemum stunt viroid (csvd), *in situ* hybridization, plasmodesmata, shoot apical meristem

Given that they do not encode proteins, viroid RNAs are extremely dependent on host factors for completing their infection cycle. This cycle consists of: (i) access to the organelle wherein they are replicated, the nucleus in the family *Pospiviroidae* (type member potato spindle tuber viroid, PSTVd) and plastids in the *Avsunviroidae* (type member avocado sunblotch viroid, ASBVd), (ii) replication, (iii) exit and cell-to-cell movement, and (iv) long-distance movement and downloading to infect distal plant parts. In addition, viroids need to circumvent the defense response mounted by their hosts (see for a review Flores et al., 2015). With respect to movement, when injected into symplasmically connected mesophyll cells of tomato and tobacco, PSTVd moves quickly from cell to cell, strongly suggesting the involvement of plasmodesmata (PD; Ding et al., 1997). The same viroid traffics long distance in the phloem of *Nicotiana benthamiana*, with this trafficking being probably sustained by replication in the phloem and by plant developmental and cellular factors (Zhu et al., 2001). The latter study also showed that PSTVd was absent from the shoot apical meristem (SAM), a finding with practical implications.

Infection by viroids is frequently accompanied by symptoms that include stunting and leaf, flower and fruit deformation and discoloration in plants of economic interest. Therefore, the use of viroid-free plants is crucial for their sustainable production. Several procedures have been implemented to generate viroid-free plants, prominent among which is shoot-tip culture or micrografting. The rationale for this procedure is most likely multiple and includes RNA silencing, as revealed by the involvement of RNA-dependent RNA polymerase 6 in delaying accumulation and precluding meristem invasion of PSTVd in *N. benthamiana* (Di Serio et al., 2010). Besides this, the relative vascular isolation of the SAM from the surrounding tissue may also hamper viroid invasion, with the fraction of regenerated viroid-free plants being dependent on the size of the shoot tip as well as on the specific viroid, host, and even cultivar. For instance, although PSTVd and the closely related tomato apical stunt viroid (TASVd) cannot invade the tomato SAM (Qi and Ding, 2003; Matsushita et al., 2011), the size of the viroid-free region adjacent to the SAM differs between the two viroids, being smaller in the case of TASVd.

Zhang et al. (2015) now report a study on another close relative of PSTVd, chrysanthemum stunt viroid (CSVd), which in certain *Argyranthemum* cultivars induces symptoms—such as yellow deformed leaves with terminal necrosis, flower distortion, or leaf necrosis—resembling those previously observed in CSVd-infected chrysanthemum. Earlier data suggest that CSVd access to meristematic tissue depends on the specific chrysanthemum cultivar examined, but no systematic study on the ability of the same viroid to invade the SAM of different cultivars of a given host plant has been performed, leaving unanswered the question of why the relative success in producing viroid-free plants using the same method varies among cultivars. Using *in situ* hybridization, histological, and immunological techniques, transmission electron microscopy and RT-PCR, the present work by Zhang et al. (2015) shows that invasion of SAM by CSVd indeed differs among *Argyranthemum* cultivars. While in cultivar “Yellow Empire” CSVd was found by *in situ* hybridization in all tissues including the uppermost cell layers of the apical dome (AD) and the two youngest leaf primordia (LP 1 and 2), in cultivar “Border Dark Red” the viroid reached the lower part of the AD and LP 3, but neither the upper part of the AD nor LP 1 and 2. Histological examinations and transmission electron microscopy revealed in both cultivars similar developmental patterns of vascular tissues and plasmodesmata (PD) in the SAM, not explaining the differences observed in the ability of CSVd to invade the SAM of *Argyranthemum* cultivars.

However, immunolocalization analyses of ultra-thin sections incubated with a monoclonal antibody against β-1, 3-glucans showed that the number of callose (β-1,3-glucan) particles deposited at PD of SAM was lower in “Yellow Empire” than in “Border Dark Red,” leading the authors to associate the observed difference to the differential ability of CSVd to invade the SAM of the two *Argyranthemum* cultivars. This intriguing conclusion is based on prior results suggesting that PD function could be regulated by callose deposition (Xu and Jackson, 2010), which would compress the plasma membrane inward and reduce the transit space. Thus, as previously suggested for viruses (Iglesias and Meins, 2000), callose deposition at PD might raise a physical barrier restricting cell-to-cell movement of viroids. Whether they move as free RNAs or associated to some host protein is not known.

It is worth noting that CSVd symptoms were observed in both *Argyranthemum* cultivars, thus discarding the involvement of callose deposition at PD of SAM in disease induction. These results are somewhat at odds with the recent claim that targeting of tomato callose synthase mRNAs by a small RNA—generated by RNA silencing from the virulence modulating region of certain PSTVd variants—could contribute to inducing the typical symptoms associated with infection (Adkar-Purushothama et al., 2015). However, care must be exerted when linking the results of both studies considering that they have been performed with different viroid-host combinations and that Zhang et al. (2015) only looked at callose deposition at the SAM. In contrast, invasion of SAM by certain variants of peach latent mosaic viroid (PLMVd; family *Avsunviroidae*) inducing albinism might play a role in pathogenesis because SAM proplastids and mature leaf plastids present rudimentary thylakoids. Furthermore, processing of plastid rRNAs is impaired in albino tissues, which has deleterious effects on the plastid translation machinery. Because all plastids derive from proplastids, which are present in the meristems, and because PLMVd replicates in albino tissues and was detected in the AD, the viroid most likely interferes with an early step of chloroplast development resulting in albino-variegated phenotypes resembling those of certain mutants in which plastid rRNA maturation is also impaired (Rodio et al., 2007). The ultimate mechanism seems to involve RNA silencing-mediated cleavage of the mRNA encoding the chloroplastic heat-shock protein 90 (cHSP90), which participates in chloroplast biogenesis (see for a review Flores et al., 2015).

## Author contributions

The author confirms being the sole contributor of this work and approved it for publication.

### Conflict of interest statement

The author declares that the research was conducted in the absence of any commercial or financial relationships that could be construed as a potential conflict of interest.
